# Efficacy of concurrent chemoradiotherapy plus Endostar compared with concurrent chemoradiotherapy in the treatment of locally advanced nasopharyngeal carcinoma: a retrospective study

**DOI:** 10.1186/s13014-022-02104-4

**Published:** 2022-07-29

**Authors:** Yuanxiu Yin, Ziyan Zhou, Zhiru Li, Mingjun Shen, Yating Qin, Chaolin Yang, Rensheng Wang, Min Kang

**Affiliations:** 1 Department of Radiation Oncology, The First Affiliated Hospital of Guangxi Medical University, No. 6, Shuangyong Road, Nanning, 530021 Guangxi China; 2 Key Laboratory of Early Prevention and Treatment for Regional High Frequency Tumor (Guangxi Medical University), Ministry of Education, Nanning, 530021 Guangxi China; 3 Guangxi Key Laboratory of Immunology and Metabolism for Liver Diseases, Nanning, 530021 Guangxi China

**Keywords:** Nasopharyngeal cancer, Lymph nodes, Concurrent chemoradiotherapy, Endostar

## Abstract

**Background:**

To retrospectively analyze the efficacy and safety of concurrent chemoradiotherapy (CCRT) plus recombinant human endostatin (Endostar, CCRT + E) versus CCRT alone in locally advanced nasopharyngeal carcinoma (LANPC).

**Methods:**

A retrospective analysis of patients initially treated for LANPC from November 2016 to March 2019 was performed: trial group received CCRT + E and control group received CCRT. Prognoses and adverse effects were evaluated.

**Results:**

Eighty-eight patients were included: 43 received CCRT + E and 45 received CCRT. The median follow-up time was 54.0 (range: 8.0–64.0) months. The survival data of the CCRT + E and CCRT groups were as follows: 3-year progression-free survival (PFS) rates, 81.4% and 63.6% (hazard ratio [HR] 0.418, 95%CI 0.181–0.963, *P* = 0.034); 3-year distant metastasis-free survival (DMFS) rates, 88.3% and 77.3% (HR 0.370, 95%CI 0.132–1.039, *P* = 0.049); 3-year overall survival rates, 88.2% and 81.9% (HR 0.437, 95%CI 0.151–1.260, *P* = 0.114); and 3-year locoregional failure-free survival rates, 87.8% and 86.9% (HR 0.795, 95%CI 0.242–2.616, *P* = 0.705). Three months after radiotherapy, the complete response (CR) rates of cervical lymph node regression were 97.7% and 82.2% for the CCRT + E and CCRT groups (*P* = 0.041). The corresponding CR rates were 100% and 80.0% for lymph node necrosis (*P* = 0.001) and 100% and 85.2% for extranodal extension (*P* = 0.041). The CCRT + E group had higher incidence of grade 3/4 leukopenia (32.6% vs. 13.3%, *P* = 0.031), with similar results for late toxicity.

**Conclusions:**

CCRT + E significantly prolonged 3-year PFS and DMFS in LANPC, and patients had better lymph node regression.

## Introduction

Cases of nasopharyngeal carcinoma (NPC) are reported throughout Southeast Asia and are common in China's Guangxi and Guangdong provinces. The annual incidence of NPC in Guangxi is 10–30 per 100,000 people, with an increased mortality rate [1, 2]. The wide use of intensity-modulated radiation therapy (IMRT) has led to an effective improvement in survival rates of patients with NPC compared with previous two-dimensional radiotherapies, with 5-year overall survival rates reaching 86.6–93.2% in stages I–II and 63.2–80.5% in stages III–IVB [3]. Currently, concurrent chemoradiotherapy (CCRT) is the standard of care for locally advanced nasopharyngeal carcinoma (LANPC), but distant metastases remain a significant cause of treatment failure [3–5]. Therefore, new therapies are needed to lower the distant metastatic rate and improve treatment efficacy in sufferers with LANPC.

Endostar, a recombinant human vascular endothelial inhibitor, is a multi-targeted tumor cell inhibitor developed in China [6]. Endostar directly inhibits vascular endothelial cell multiplication and suppresses tumor development through multiple targets, including vascular endothelial growth factor (VEGF), VEGF receptor-2 (VEGFR-2), and platelet-derived growth factor receptor [7]. It can also normalize tumor blood vessels and exert antitumor effects [7, 8]. Lymphatic metastasis is common in most malignancies, including NPC. The generation of lymphatic vessels provides a pathway for neoplastic cells to reach sentinel lymph nodes and distant organs [9, 10]. Recombinant human vascular endothelial inhibitors inhibit tumor lymphatic vessel production and lymphatic metastasis by inhibiting the expression of VEGF-C and VEGF-D in tumor tissues, decreasing the activation of VEGFR-3 in tissues suppressing the VEGF-C and VEGF-D/VEGFR-3 signal pathways [11]. Zhou et al. [12] established a mouse model of NPC and found that Endostar radiosensitized NPC by decreasing VEGF expression. Phase II clinical trials have confirmed that Endostar plus chemotherapy is safe and effective in treating nasopharyngeal and head and neck squamous carcinoma [13–15].

Therefore, this study aimed to retrospectively analyze the efficacy and safety of CCRT plus recombinant human endostatin (Endostar, CCRT + E) versus CCRT alone in LANPC.

## Materials and methods

### Patients

Ninety-six patients with LANPC treated at the First Affiliated Hospital of Guangxi Medical University, China, from November 2016 to March 2019 were reviewed. However, seven patients were excluded for not having cervical lymph node metastases and one for not completing radiotherapy. Finally, 88 patients were included: 43 were treated with CCRT + E (trial group), and 45 received CCRT (control group).

The following criteria were met: (a) pathologically confirmed nasopharyngeal carcinoma; (b) untreated stage III–IVb patients with stages T1–2N2, T3–4N1–2, or T1–4N3 (according to the Union for International Cancer Control [UICC] 7th edition staging system); (c) no evidence of distant metastasis; (d) aged 18–70 years; (e) normal hematologic function; f) normal liver and renal functions, and (g) Karnofsky performance status of ≥ 70 points. The exclusion criteria were as follows: (a) previous radiotherapy, chemotherapy, or targeted therapy for nasopharyngeal cancer; (b) recurrence or distant metastasis; (c) T1–2N0–1 or T3–4N0 (UICC 7th edition staging system); (d) previous malignancy; (e) uncontrolled life-threatening disease; and (f) pregnancy or lactation.

The medical history of the patient was collected before treatment. A physical examination was performed, including nasopharyngeal fiber endoscopy, head and neck enhanced magnetic resonance imaging (MRI), enhanced computed tomography (CT, only for patients for whom MRI was prohibited), chest CT, and liver imaging (abdominal ultrasound or CT), bone imaging, and electrocardiogram. Additionally, a blood sample was collected. Positron emission tomography CT (PET/CT) was optional.

### Treatments

#### Endostar

Endostar (7.5 mg/m^2^/day, days 1–10) was administered by continuous intravenous pumping from 5 days before radiotherapy for 10 consecutive days for three cycles. From the first day after the completion of radiotherapy, the patients received another two cycles of Endostar at an interval of 7 days.

#### Concurrent chemoradiotherapy

In both groups, the target area and dose design of IMRT for NPC, according to reports 50 and 62 of the International Radiation Unit and Measurement Organization (ICRU) and the expert consensus of the Radiation Treatment Oncology Organization (RTOG) 0225. GTVnx includes primary NPC foci and enlarged retropharyngeal lymph nodes, whereas GTVnd includes imaging and palpation findings of enlarged cervical lymph nodes. The high-risk clinical target volume (CTV1) was a 5–10 mm outward expansion of the GTVnx (or 1–3 mm if close to the brainstem or spinal cord) to cover the submicroscopic increase in the high-risk site and entire nasopharynx. The low-risk clinical target volume (CTV2) was a 5–10 mm outward expansion of CTV1 to include the skull base, alveolus, cavernous sinus, parapharyngeal space, pterygopalatine fossa, posterior nasal cavity, and the lymph node drainage area in the neck. For the planned target regions derived from GTVnx, GTVnd, CTV1, and CTV2, the administered doses were 70–72, 64–72, 62–64, and 54–56 Gy, respectively, 30–33 f, five times a week for 6–7 weeks; concurrent chemotherapy regimens included cisplatin (80 mg/m^2^), nedaplatin (80 mg/m^2^), and docetaxel plus cisplatin (DOC 75 mg/m^2^, DDP 80 mg/m^2^). A cycle included 21 days.

#### Follow-up

After the completion of radiotherapy, assessments were performed every 3 months for 1–3 years, every 6 months for 4–5 years, and annually thereafter. The review included head and neck enhanced MRI or enhanced CT (for patients for whom MRI was prohibited), chest CT, liver imaging (abdominal ultrasound or CT), bone imaging, nasopharyngoscopy, and laboratory examination. PET/CT was optional.

Efficacy was evaluated according to solid tumor version 1.1 (RECIST1.1), and efficacy indicators included complete response (CR), partial response, stable disease, and progressive disease. Toxic reactions were evaluated according to the National Cancer Institute Common Terminology Criteria for Adverse Events (NCI-CTCAE v4.0).

### Statistical analysis

Statistical analyses were performed using SPSS Statistics (version 25.0; IBM Corp., Armonk, NY, USA) and GraphPad Prism 6.0. *P* < 0.05 was considered statistically significant. The χ^2^ test or Fisher's exact test was used to detect qualitative variables and adverse effects, and differences in continuous variables were assessed using the Mann–Whitney *U* test. Survival curves were analyzed using the Kaplan–Meier method. The hazard ratios (HRs) were analyzed using the Cox proportional hazard model.

## Results

### Patient characteristics

Our study included 88 patients with initially treated LANPC, of whom 61 (69.3%) were men. The mean age of patients in both groups was 44.2 years (range: 24–66). The number of patients with clinical stages III, IVa, and IVb was 31, 46, and 11, respectively. The number of patients with lymph node stages of N1, N2, and N3 was 45, 26, and 11, respectively. The sex, tumor (T) stage, clinical stage, lymph node (N) stage, and age were similar in the two groups (*P* > 0.05) (Table 1).

**Table 1:** Clinical characteristics of the two groups

	CCRT + E group (n = 43)	CCRT group (n = 45)	*P* value
Average age (range)	44.7 years (27–64)	43.6 years (24–66)	0.515
Sex			
Men	29 (67.4%)	32 (71.1%)	0.709
Women	14 (32.6%)	13 (28.9%)	
Clinical stage (7th,UICC/AJCC)			0.955
III	16 (37.2%)	15 (33.3%)	
IVa	22 (51.2%)	24 (53.3%)	
IVb	5 (11.6%)	6 (13.3%)	
T stage			0.609
T1	0 (0)	0 (0)	
T2	2 (4.7%)	5 (11.1%)	
T3	16 (37.2%)	15 (33.3%)	
T4	25 (58.1%)	25 (55.6%)	
N stage			0.312
N0	0 (0)	0 (0)	
N1	21 (48.8%)	28 (62.2%)	
N2	17 (39.5%)	11 (24.4%)	
N3	5 (11.6%)	6 (13.3%)	

CCRT, concurrent chemoradiotherapy; E, Endostar; T, primary tumor; N, regional lymph nodes

During concurrent chemoradiotherapy, all 43 (100%) patients in the CCRT + E group completed three processes of Endostar. After radiotherapy, 37 (86.0%) patients received two cycles of Endostar, and four (9.3%) received one cycle of Endostar. Two (4.7%) patients did not continue Endostar maintenance treatment. All 43 (100%) patients received ≥ 2 cycles of concurrent chemotherapy, of whom 40 (93.0%) were on single-agent cisplatin regimens, and three (7.0%) received both single-agent cisplatin and single-agent nedaplatin regimens. In the CCRT group, 43 patients (95.6%) received ≥ 2 cycles of chemotherapy and two (4.4%) received one cycle. Of them, 39 (86.7%) patients were treated with cisplatin alone, one (2.2%) received only docetaxel plus cisplatin, and four (8.9%) received both single-agent cisplatin and docetaxel plus cisplatin, and one (2.2%) received all three regimens. Cisplatin dose was reduced in four CCRT + E and three CCRT patients due to hematological toxicity reactions and gastrointestinal reactions, respectively. In the CCRT + E group, one patient was suspended from radiotherapy for 12 days due to infectious shock, and the dose of radiotherapy was increased to GTVnx 77 Gy/35 f. The remaining patients completed the radiotherapy program according to the original protocol.

### Tumour response and survival analysis

Three months after radiotherapy, the CR rates of nasopharyngeal lesions were 53.5% (23/43) and 60.0% (27/45) in the CCRT + E and CCRT groups, respectively. The recent outcomes of nasopharyngeal lesions were similar in both groups (*P* = 0.538) (Table 2).

**Table 2:** Response of the nasopharyngeal lesions and lymph node lesions

	CCRT + E	CCRT	*P* value for CR rate
Nasopharyngeal lesions			
CR	23 (53.5%)	27 (60.0%)	0.538
PR	20 (46.5%)	18 (40.0%)	
SD	0 (0)	0 (0)	
Lymph node lesions			
CR	42 (97.7%)	37 (82.2%)	0.041
PR	1 (2.3%)	8 (17.8%)	
SD	0 (0)	0 (0)	

CR, complete remission; PR, Partial remission; SD, stable disease

The lymph node CR rates were 97.7% (42/43) and 82.2% (37/45) in the CCRT + E and CCRT groups, respectively (*P* = 0.041) (Table 2), and the result suggested that patients in the CCRT + E group had better overall lymph node regression than the CCRT group. There were 104 and 113 measurable regional lymph nodes in the two groups, respectively. The CR rates of retropharyngeal lymph nodes in patients in the CCRT + E and CCRT groups were 95.5% (42/44) and 84.4% (38/45), respectively (*P* = 0.170). The CR rates of lymph node necrosis were 100% (47/47) and 80.0% (40/50), respectively (*P* = 0.001). The CR rates of extranodal extension of the lymph nodes were 100% (31/31) and 85.2% (23/27), respectively (*P* = 0.041). Lymph node necrosis and extranodal extension of the lymph nodes were associated with better outcomes in the CCRT + E group. The CR rates of lymph nodes in the CCRT + E and CCRT groups were 97.8% (89/91) and 88.9% (88/99) in the subgroup of lymph nodes with the shortest diameter ≤ 3 cm, respectively (*P* = 0.015), and 100% (13/13) versus 71.4% (10/14) in the subgroup of lymph nodes with the shortest diameter > 3 cm, respectively (*P* = 0.098) (Table 3).

**Table 3:** Response of subgroups of lymph nodes

	CCRT + E	CCRT	*P* value for CR rate
Retropharyngeal lymph nodes			
CR	42 (95.5%)	38 (84.4%)	0.170
PR	2 (4.5%)	6 (13.3%)	
SD	0 (0)	1 (2.2%)	
Lymph nodal necrosis			
CR	47 (100%)	40 (80.0%)	0.001
PR	0 (0)	10 (20.0%)	
SD	0 (0)	0 (0)	
Extranodal extension of lymph nodes			
CR	31 (100%)	23 (85.2%)	0.041
PR	0 (0)	4 (14.8%)	
SD	0 (0)	0 (0)	
Shortest diameter ≤ 3 cm of lymph nodes			
CR	89 (97.8%)	88 (88.9%)	0.015
PR	2 (2.2%)	8 (8.1%)	
SD	0 (0)	3 (3.0%)	
Shortest diameter > 3 cm of lymph nodes			
CR	13 (100.0%)	10 (71.4%)	0.098
PR	0 (0)	4 (28.6%)	
SD	0 (0)	0 (0)	

CR, complete remission; PR, partial remission; SD, stable disease

**Fig. 1:**
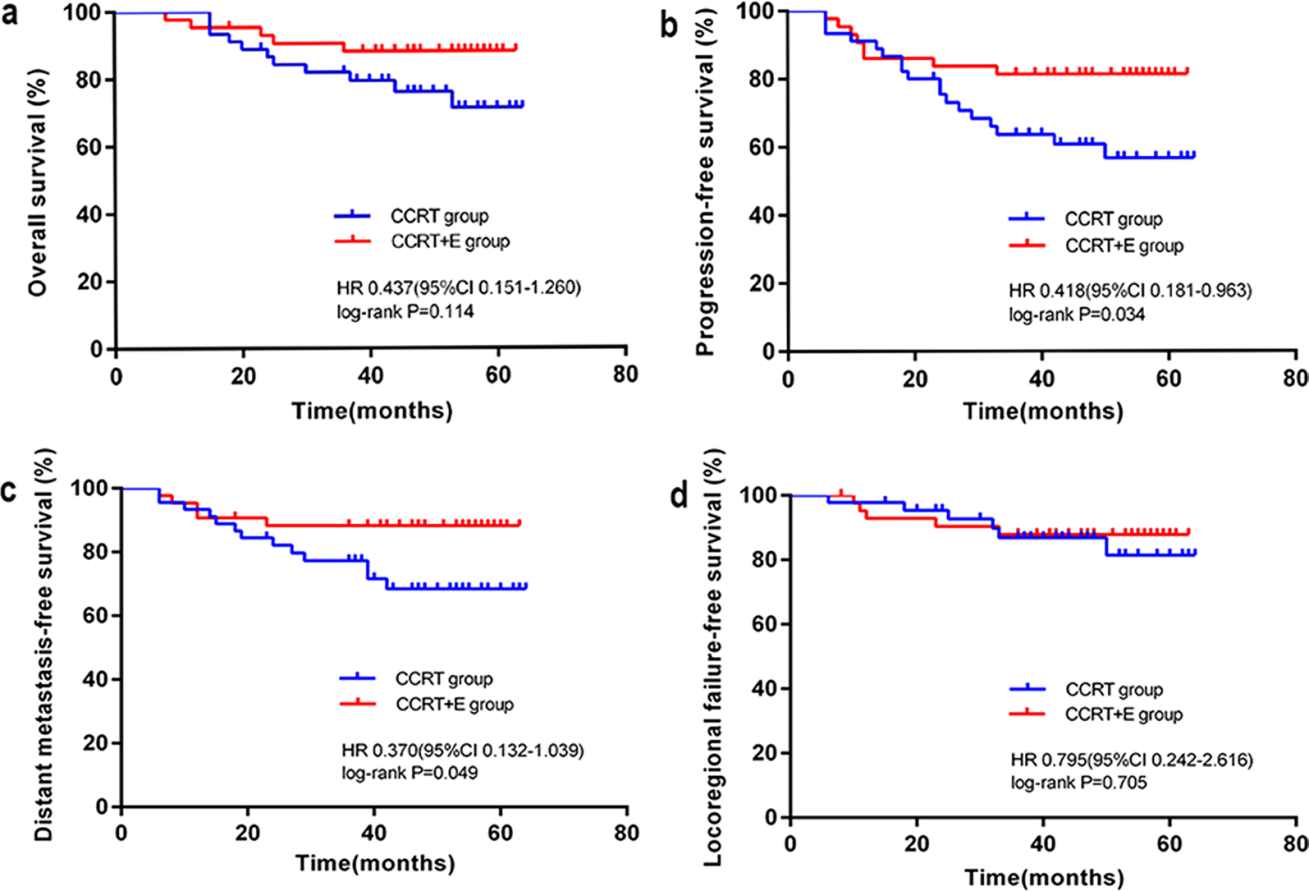
Cumulative survival curves after treatment with concurrent chemoradiotherapy plus Endostar compared with concurrent chemoradiotherapy. **a** Overall survival; **b** Progression-free survival; **c** Distant metastasis-free survival; **d** Locoregional failure -free survival. HR, hazard ratio; CI, confidence interval

There were 88 patients in both groups, with a median follow-up time of 54.0 (range: 8.0–64.0) months. There were five (11.6%) deaths in the CCRT + E group and 10 (22.2%) in the CCRT group (Table 4), all of which were nasopharyngeal cancer-related deaths. In the CCRT + E group, five (11.6%) patients had distant metastases: four in the lungs, two in the liver, and four in the bone. Four of these patients presented with ≥ 2 organ metastases. Thirteen (28.9%) patients in the CCRT group had distant metastases: six cases of lung metastasis, three of liver metastasis, seven of bone metastasis, and two of other sites. Six of these patients presented with ≥ 2 organ metastases (Table 4). Locoregional recurrence occurred in five (11.6%) patients in the CCRT + E group and six (13.3%) patients in the CCRT group (Table 4). The 3-year overall survival (OS), progression-free survival (PFS), distant metastasis-free survival (DMFS), and locoregional failure-free survival (LRFFS) in CCRT + E and CCRT were 88.2% versus 81.9% (hazard ratio [HR] 0.437, 95% CI 0.151–1.260; log-rank *P* = 0.114), 81.4% versus 63.6% (HR 0.418, 95% CI 0.181–0.963; log-rank *P* = 0.034), 88.3% versus 77.3% (HR 0.370, 95% CI 0.132–1.039; log-rank *P* = 0.049), and 87.8% versus 86.9% (HR 0.795, 95%CI 0.242–2.616; log-rank *P* = 0.705), respectively (Fig. 1).

**Table 4:** Patterns of disease failure in patients treated with CCRT + E versus CCRT

Failure pattern	CCRT + E (n = 43)	CCRT (n = 45)	*P* value
Locoregional relapse only	3 (7.0%)	4 (8.9%)	1.000
Distant metastases only	3 (7.0%)	7 (15.6%)	0.352
Both locoregional relapse and distant metastases	2 (4.7%)	3 (6.7%)	1.000
Metastatic sites			
Bone	4 (9.3%)	7 (15.6%)	0.375
Liver	2 (4.7%)	3 (6.7%)	1.000
Lung	4 (9.3%)	6 (13.3%)	0.795
Other	0 (0)	2 (4.4%)	0.495
Death	5 (11.6%)	10 (22.2%)	0.186

### Adverse effects

Acute adverse events during treatment in the CCRT + E and CCRT groups are shown in Table 5. Grade 3 or 4 adverse reactions were mainly focused on leukopenia, thrombocytopenia, decreased hemoglobin, nausea, vomiting, dry mouth, oral mucositis, and radiation dermatitis. The incidence of grade 3 or 4 leukopenia was significantly higher in the CCRT + E group (14/43, 32.6%) than in the CCRT group (6/45, 13.3%) (*P* = 0.031), which improved after symptomatic treatment without treatment interruption or cessation. The incidence of other grade 3 or 4 adverse reactions was similar in both groups (*P* > 0.05). The negative effects of grade 1 or 2 in both groups were leukopenia, decreased hemoglobin, weight loss, nausea, vomiting, dry mouth, oral mucositis, liver dysfunction, renal dysfunction, and skin reactions; these events were not significantly different between the groups (*P* > 0.05).

**Table 5:** Acute toxic reactions

Adverse event	CCRT + E (n = 43)			CCRT (n = 45)			*P* value for events Grade 1 and 2	*P* value for events Grade 3 and 4
	1 + 2	3	4	1 + 2	3	4		
Leucopenia	28 (65.1)	14 (32.6)	0 (0)	34 (75.6)	5 (11.1)	1 (2.2)	0.283	0.031
Thrombocytopenia	8 (18.6)	2 (4.7)	0 (0)	4 (8.9)	0 (0)	0 (0)	0.309	0.236
Hemoglobin decrease	26 (60.5)	3 (7.0)	0 (0)	22 (48.9)	1 (2.2)	0 (0)	0.276	0.355
Weight loss	30 (69.8)	0 (0)	0 (0)	36 (80.0)	0 (0)	0 (0)	0.268	–
Nausea	38 (88.4)	1 (2.3)	0 (0)	41 (91.1)	1 (2.2)	0 (0)	0.943	1.000
Vomiting	29 (67.4)	2 (4.7)	0 (0)	34 (75.6)	2 (4.4)	0 (0)	0.399	1.000
Dry mouth	43 (100.0)	0 (0)	0 (0)	42 (93.3)	2 (4.4)	0 (0)	0.242	0.495
Diarrhea	3 (7.0)	0 (0)	0 (0)	2 (4.4)	0 (0)	0 (0)	0.958	–
Constipation	12 (27.9)	0 (0)	0 (0)	11 (24.4)	0 (0)	0 (0)	0.712	–
Oral mucositis	39 (90.7)	1 (2.3)	0 (0)	41 (91.1)	2 (4.4)	0 (0)	1.000	1.000
Dermatitis	40 (93.0)	2 (4.7)	0 (0)	42 (93.3)	1 (2.2)	0 (0)	1.000	0.612
Liver dysfunction	13 (30.2)	0 (0)	0 (0)	11 (24.4)	0 (0)	0 (0)	0.542	–
Renal dysfunction	13 (30.2)	0 (0)	0 (0)	9 (20.0)	0 (0)	0 (0)	0.268	–
Cardiac dysfunction	0 (0)	0 (0)	0 (0)	0 (0)	0 (0)	0 (0)	–	–

Data are n (%)

None of the grade 3 or 4 late toxic reactions occurred in the CCRT + E group. In the CCRT group, two patients had grade 3 hearing impairment; however, no grade 4 adverse reactions occurred. Limitation of mouth opening, decreased vision, hearing loss, temporal lobe damage, subcutaneous fibrosis, cranial nerve palsy, and dry mouth were the major adverse effects of grade 1 or 2, with similar incidence in both groups (*P* > 0.05) (Table 6).

**Table 6:** Late toxic reactions

Adverse event	CCRT + E (n = 43)			CCRT (n = 45)			*P* value for events Grade 1 and 2	*P* value for events Grade 3 and 4
	1 + 2	3	4	1 + 2	3	4		
Limitation of mouth opening	1 (2.3)	0 (0)	0 (0)	1 (2.2)	0 (0)	0 (0)	1.000	–
Decreased vision	1 (2.3)	0 (0)	0 (0)	2 (4.4)	0 (0)	0 (0)	1.000	–
Hearing loss	6 (14.0)	0 (0)	0 (0)	6 (13.3)	2 (4.4)	0 (0)	0.932	0.495
Temporal lobe necrosis	1 (2.3)	0 (0)	0 (0)	1 (2.2)	0 (0)	0 (0)	1.000	–
Subcutaneous fibrosis	1 (2.3)	0 (0)	0 (0)	0 (0)	0 (0)	0 (0)	0.489	–
Dry mouth	5 (11.6)	0 (0)	0 (0)	3 (6.7)	0 (0)	0 (0)	0.661	–
Cranial nerve palsy	3 (7.0)	0 (0)	0 (0)	2 (4.4)	0 (0)	0 (0)	0.958	–
Cardiac dysfunction	0 (0)	0 (0)	0 (0)	0 (0)	0 (0)	0 (0)	–	–

Data are n (%)

## Discussion

This retrospective study showed that CCRT + E is effective in reducing the rate of distant metastases and promoting lymph node regression in patients with LANPC. Specifically, CCRT + E significantly improved 3-year DMFS and PFS in patients with LANPC and improved CR rates in cervical lymph nodes, including lymph node necrosis and extranodal extension.

Currently, distant metastasis remains the leading cause of treatment failure in NPC [3–5, 16], and a similar result was found in this study, with 17% of patients experiencing distant metastasis at 3 years. This result implies that reducing the rate of distant metastasis is the key to improving the outcome of NPC. Previous studies have shown that blood and lymphatic vessels influence the oncogenesis, development, and metastasis of neoplasms, indicating that inhibiting the production of tumor blood and lymphatic vessels can impede the development and metastasis of neoplasms [17, 18]. VEGF is a tumor-inducing factor that contributes to angiogenesis [19, 20] and is an adverse prognostic factor in patients with NPC [21, 22]. Endostar restrains tumor development and lymph node metastasis by suppressing VEGF expression, inhibiting tumor vascular and lymphatic vessel production, and reducing the entry of tumor cells into circulation [7]. As confirmed in this study, CCRT + E significantly improved 3-year DMFS and PFS in patients with LANPC. In the study by Li et al. [15], the CR rates of cervical lymph nodes in the CCRT + E and CCRT groups were 91.1% and 71.4%, respectively, with a significant difference (*P* = 0.048), consistent with the result of the present study (97.7% vs. 82.2%, *P* = 0.041).

The 3-year PFS of patients in the CCRT group in this study was 63.6%, and 3-year DMFS was 77.3%, which was similar to the results of previous studies [23, 24]. In a phase II clinical study by Fountzilas et al. [23], the 3-year PFS rate in LANPC patients receiving CCRT was 63.5%. A phase II–III randomized clinical study by Tan et al. [24] reported 3-year DFS and DMFS rates of 67.4% and 79.9%, respectively. In a study by Li et al. [25] in which sequential Endostar plus induction chemotherapy and concurrent chemoradiotherapy were compared with sequential induction chemotherapy and concurrent chemoradiotherapy for LANPC, the 2-year OS, PFS, and DMFS rates in the trial group were 82.3%, 77.2%, and 82.2%, respectively. Here, the 3-year OS, PFS, and DMFS (88.2%, 81.4%, and 88.3%, respectively) were higher than the 2-year survival rates reported by Li et al. [25], wherein the trial group received only one cycle of Endostar in concurrent chemoradiotherapy. In contrast, in this study, the trial group (of 43 patients) received three cycles of Endostar during concurrent chemoradiotherapy—this could explain the observed differences in survival. It has been shown that Endostar is effective in inducing vascular normalization 5–7 days after administration, and when administered in combination, two consecutive cycles of treatment are required to assess efficacy [26–28]. Endostar increases radiotherapy sensitivity [12, 29], and increasing the dosing cycle of Endostar during concurrent chemoradiotherapy improves radiotherapy sensitivity and thus its efficacy.

Approximately 85% of patients initially diagnosed with nasopharyngeal carcinoma have cervical lymph node metastases [30]. Cervical lymph node metastases are closely related to patient survival prognosis, and lymph node necrosis and extranodal extension are independent prognostic factors of NPC [31–33]. Hence, improving lymph node outcomes is crucial. The results of this study showed that 3 months after radiotherapy, the CCRT + E group had better outcomes for lymph node necrosis (*P* = 0.001), lymph nodes of extranodal extension (*P* = 0.041), and lymph nodes with the shortest diameter ≤ 3 cm (*P* = 0.015). The reason for the enhanced therapeutic effect of the CCRT + E regimen could be that Endostar inhibits the production of tumor blood vessels and lymphatic vessels and induces the normalization of tumor blood vessels, thereby improving tumor cell hypoxia and enhancing the radiosensitivity of hypoxic cells. The CR rate of lymph nodes with the shortest diameters of > 3 cm was significantly better in the CCRT + E group than that in the CCRT group (100% vs. 71.4%). Nevertheless, the difference was not statistically significant (*P* = 0.098), possibly because the sample size of this study was limited. Therefore, a study with a larger sample is needed to further clarify the efficacy of CCRT + E on lymph nodes with the shortest diameters > 3 cm.

The incidence of grade 3 or 4 leukopenia in CCRT + E is highly variable across studies, ranging from 5.3 to 44.8% [15, 34–36], which may be related to different chemotherapy regimens and cycles, Endostar cycles, and radiotherapy doses. In this study, the incidence of grade 3/4 leukopenia in the CCRT + E group was 32.6%, but all patients recovered with symptomatic management, and no patient interrupted or discontinued treatment, and no patient died of acute toxic events. The late toxic reactions were similar in both groups (*P* > 0.05), indicating that CCRT + E was safe and feasible for the treatment of LANPC.

Our study had some limitations. This study was retrospective in nature, with a follow-up period of only 3 years and a limited number of patient cases. Therefore, a large prospective study observing the long-term prognosis is needed for further validation.

## Conclusions

This study showed that CCRT + E improved 3-year PFS and DMFS in patients with LANPC compared with CCRT and that patients treated with CCRT + E had better cervical lymph node regression with a manageable safety profile.

## Data Availability

All data generated and analyzed during this study are included in this published article.

## Abbreviations

CCRT: Concurrent chemoradiotherapy
E: Endostar
NPC: Nasopharyngeal carcinoma
LANPC: Locally advanced nasopharyngeal carcinoma
IMRT: Intensity-modulated radiation therapy
HR: Hazard ratio
T: Tumor
N: Lymph node
CR: Complete response
VEGF: Vascular endothelial growth factor
UICC: Union for International Cancer Control
MRI: Magnetic resonance imaging
CT: Computed tomography
PET/CT: Positron emission tomography CT
ICRU: International Radiation Unit and Measurement Organization
RTOG: Radiation treatment oncology organization
CTV: Clinical target volume
RECIST1.1: Response evaluation criteria in solid tumours 1.1
NCI-CTCAE: National Cancer Institute Common Terminology Criteria for Adverse Events
OS: Overall survival
PFS: Progression-free survival
DMFS: Distant metastasis-free survival
LRFFS: Locoregional failure-free survival
